# Fully automated determination of robotic pedicle screw accuracy and precision utilizing computer vision algorithms

**DOI:** 10.1007/s11701-024-02001-w

**Published:** 2024-07-03

**Authors:** Benjamin N. Groisser, Ankush Thakur, Howard J. Hillstrom, Akshitha Adhiyaman, Colson Zucker, Jerry Du, Matthew Cunningham, M. Timothy Hresko, Ram Haddas, John Blanco, Hollis G. Potter, Douglas N. Mintz, Ryan E. Breighner, Jessica H. Heyer, Roger F. Widmann

**Affiliations:** 1 Hospital for Special Surgery, 535 East 70th Street, New York, NY 10021 USA; 2 Boston Children’s Hospital, Boston, MA USA; 3 University of Rochester Medical Center, Rochester, NY USA

**Keywords:** Accuracy, Pedicle screw, Robotic navigation, Automated computer algorithm

## Abstract

Historically, pedicle screw accuracy measurements have relied on CT and expert visual assessment of the position of pedicle screws relative to preoperative plans. Proper pedicle screw placement is necessary to avoid complications, cost and morbidity of revision procedures. The aim of this study was to determine accuracy and precision of pedicle screw insertion via a novel computer vision algorithm using preoperative and postoperative computed tomography (CT) scans. Three cadaveric specimens were utilized. Screw placement planning on preoperative CT was performed according to standard clinical practice. Two experienced surgeons performed bilateral T2–L4 instrumentation using robotic-assisted navigation. Postoperative CT scans of the instrumented levels were obtained. Automated segmentation and computer vision techniques were employed to align each preoperative vertebra with its postoperative counterpart and then compare screw positions along all three axes. Registration accuracy was assessed by preoperatively embedding spherical markers (tantalum beads) to measure discrepancies in landmark alignment. Eighty-eight pedicle screws were placed in 3 cadavers’ spines. Automated registrations between pre- and postoperative CT achieved sub-voxel accuracy. For the screw tip and tail, the mean three-dimensional errors were 1.67 mm and 1.78 mm, respectively. Mean angular deviation of screw axes from plan was 1.58°. For screw mid-pedicular accuracy, mean absolute error in the medial–lateral and superior–inferior directions were 0.75 mm and 0.60 mm, respectively. This study introduces automated algorithms for determining accuracy and precision of planned pedicle screws. Our accuracy outcomes are comparable or superior to recent robotic-assisted in vivo and cadaver studies. This computerized workflow establishes a standardized protocol for assessing pedicle screw placement accuracy and precision and provides detailed 3D translational and angular accuracy and precision for baseline comparison.

## Introduction

Accurate and precise placement of pedicle screws is foundational to spinal fusion and spinal deformity correction; the use of pedicle screw fixation has improved both fusion rates and surgical correction [1–3]. Erroneously placed pedicle screws may result in patient morbidity and mortality, unplanned return to the operating room (UPROR), worse patient-reported outcomes and increased cost of care [4]. Recent studies report UPROR rates of 0.26–1.1% for malpositioned screws, which represents only a small subset of malpositioned screws [5–9].

Studies of pedicle screw accuracy have historically utilized review of computed tomography (CT) scans to assess and measure pedicle breach [10, 11]. The Gertzbein–Robbins (G–R) classification system defines breach as: Grade A, screw placed without breaching the cortex of the pedicle; Grade B, cortical breach < 2 mm; Grade C, cortical breach ≥ 2 mm but < 4 mm; Grade D, cortical breach ≥ 4 mm but < 6 mm; and Grade E, cortical breach ≥ 6 mm [12]. The G–R system has been the clinical “gold standard” for assessing screw safety and breach on postoperative CT [11, 13–17]. Some limitations of the G–R classification include: (1) the influence of surgeon/observer bias; (2) undetermined intrarater/interrater reliability; (3) the effect of metal-induced scatter artifact from the screws; (4) no defined standardized CT view (axial vs. coronal vs. sagittal) for assessment; (5) a lack of external validation.

The advent of computer-assisted surgical navigation has fostered development of an engineering approach to defining pedicle screw accuracy (technical accuracy) by comparing the preoperative screw plan to the actual screw position, and utilizing a Cartesian coordinate system for the reporting of absolute Euclidean error (straight-line distance in three-dimensional [3D] space) [11, 14–21]. However, a major limitation of the methodologies used in these studies is the need for manual alignment of vertebral bodies between pre- and postoperative images, introducing additional variance to the measured accuracy [14, 16, 19–33]. Using the term “accuracy” to refer to both anatomic safety measurements (i.e., G–R classification) and plan vs. placement errors results in confusion for readers attempting to evaluate best surgical practice.

The purpose of this study is to present a technically rigorous, clinically useful, and fully automated systematic approach to measure pedicle screw accuracy. This technique eliminates the need for manual alignment of the preoperative and postoperative CTs. Furthermore, we show that a screw-aligned reference frame enables us to generate detailed and clinically meaningful measurements of systematic error and precision. The emphasis on technical accuracy using continuous variables for positional and angular data facilitates direct comparisons between, and iterative improvements to, surgical navigation systems.

## Methods

### Surgery and imaging

This institutional review board-approved study utilized three adult cadavers. Cadavers were excluded if they had severe metabolic bone disease (e.g., osteoporosis), injury that would compromise the vertebrae, or spinal instrumentation.

Preoperative whole-spine helical CT scans were acquired using a GE Discovery CT750 64-slice CT scanner (GE Healthcare, Chicago, IL). The imaging protocol used 140 kVp and variable tube current (305-485 mA). Slice thickness and spacing were 0.625 mm, with pitch factor of 0.516. Reconstruction diameter (FOV) varied (range 20–30 cm) to accommodate cadaver positioning and spinal curvature, resulting in voxel sizes of 0.39 × 0.39 × 0.625 mm^3^ to 0.59 × 0.59 × 0.625 mm^3^. Screw planning was performed using Mimics (Materialize NV, Belgium), a system- and vendor-agnostic, general-purpose medical image visualization and processing software. Bilateral screws T2–L4 were planned using standard criteria to avoid medial, superior, and inferior breach. Planned screw positions were transferred into the robotic trajectory planning environment (Mazor X Stealth, Medtronic, USA).

The cadavers were dissected using a posterior midline approach with subperiosteal exposure from the spinous processes to the tips of the transverse processes. Two surgeons performed pedicle instrumentation using robotic-assisted navigation. Pedicles were drilled through the robotic end-effector, and then 4.5 mm poly-axial pedicle screws of predetermined length were placed through the end-effector.

Postoperative CT scans were performed using the similar protocols as preoperative scans, with the addition of a metal artifact reduction reconstruction. Postoperative CTs were graded by a third senior orthopedic surgeon and a senior radiologist using the Gertzbein–Robbins system. CTs were graded by creating multiplanar reconstruction and adjusting the plane of imaging to align with the pedicle screws at each level. Finally, postoperative laminectomies were conducted by skeletonizing the pedicles to assess superior, inferior, and medial breaches.

### Automated processing

**Fig. 1:**
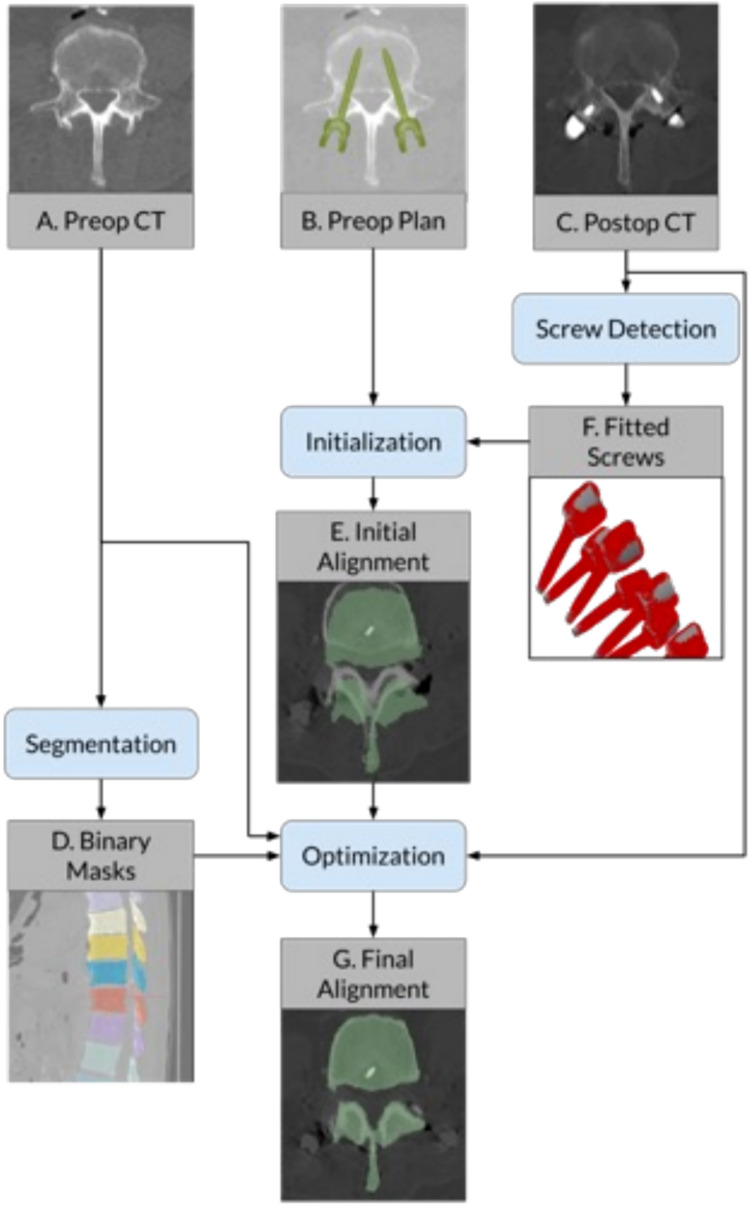
Registration workflow diagram

Using automated segmentation software, voxel-wise labels were generated for all preoperatively scanned vertebrae, defining the cortex of each bone [34]. Postoperative screw positions were determined by optimizing a poly-axial screw model to fit the CT image data. To compare preoperative plans with postoperative screw placement, automatic rigid-body alignments for each level were performed (Fig. 1).

**Fig. 2:**
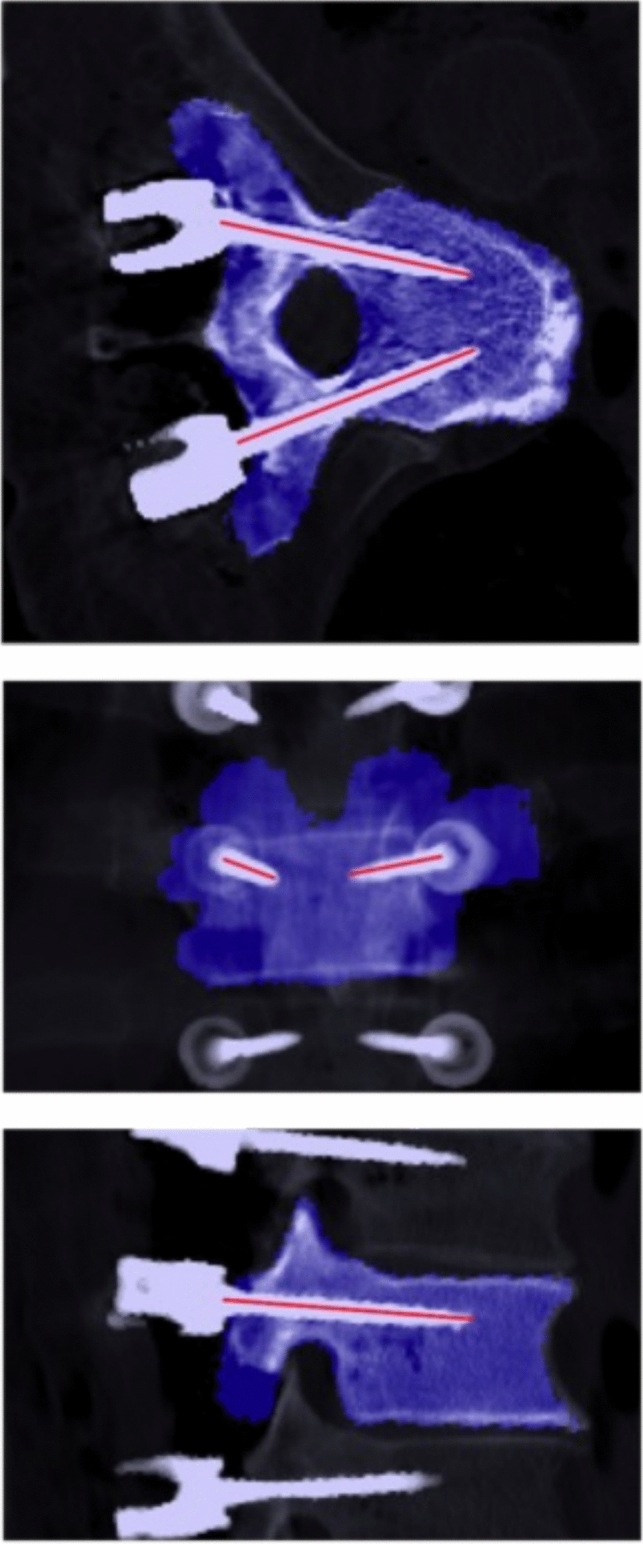
Visualization of outputs from automated CT analysis pipeline. Blue highlights show the preoperative segmentation mask registered on top of the postoperative CT. Red lines show the axes of the detected screws. Successful registration and correct identification of the screw positions indicate that the resulting accuracy measurements will be valid. Note that each image is a stacked average of several slices to visualize the screws across multiple planes

Registrations for each vertebral level were initialized using singular value decomposition to align preoperatively planned screw tip and tail landmarks with corresponding points on the detected screws, then optimized by maximizing Mutual Information between voxel intensity of pre- and postoperative CT volumes (Fig. 2) [35, 36]. The optimization step was performed using the diffusion imaging in Python package; segmentation labels were used as inclusive masks for the preoperative volumes while screws were excluded from the postoperative volumes by an intensity threshold [37].

These rigid-body registrations were then applied to map the planned screw trajectories into the postoperative image space. Screw accuracy was measured as translational and angular deviations along all three axes. The entire analysis pipeline (vertebral segmentation, screw detection, image alignment, and error measurements) was fully automated to ensure the objectivity and repeatability of the protocol. Outside of open source tools, all analyses were performed with custom Python code [34, 37].

To test the accuracy of the automated registrations, a total of 16 1 mm tantalum beads were inserted percutaneously across 4 vertebral levels in an equivalently instrumented cadaveric specimen, age 71 years. These beads were manually identified in pre- and postoperative CT and positions were compared after rigid-body alignment (beads being masked out during registration). Perturbation analysis was performed to test registration convergence: initializations for each of the 45 levels involved in the screw analysis were perturbed by 10 random rigid transforms of 2° and 2 mm and the resulting registrations compared to the unperturbed baseline registration.

### Coordinate system

Error analysis for each screw was performed in a reference frame oriented to the planned screw trajectory (Fig. 2). For pedicle screws, coordinate axes were approximately aligned with the radiographic right anterior–superior (RAS) standard. For ease of interpretation, we refer to these axes as medial–lateral (ML), anterior–posterior (AP), and superior–inferior (SI). The AP axis was aligned to the planned screw shaft. Next, we fit a plane to the inferior vertebral endplate; the SI axis is defined as the portion of that plane’s normal perpendicular to the AP axis. The ML axis is orthogonal to these two axes.

Accuracy measurements were performed at the screw tip and tail, as well as the “mid pedicle,” defined as the point along the pedicle where the screw comes closest to (or maximally breaches) the medial pedicle wall. All analyses of right sided screws are illustrated as mirrored across the sagittal plane, allowing direct comparison across all screws while maintaining anatomically relevant directionality.

### Statistical analysis

Group-level angular and translation errors are expressed as mean absolute error (MAE) and signed mean error (SME, or arithmetic mean), and 2 standard deviations (SD). SD is computed on the raw (signed) errors and represents the “spread” or consistency of screw placement, i.e., precision. Clinical safety is described by breach rates as well as the G–R grade. One-way ANOVA and post hoc multiple comparison tests with Bonferroni correction were done to test for accuracy differences in the upper thoracic (T2–T6), lower thoracic (T7–T12), and lumbar (L1–L4) regions. Statistical tests were completed using SPSS version 29 (IBM Corp, Armonk, NY).

## Results

Ninety pedicle screws were planned in 3 cadavers, aged 54, 69 and 84 years. Eighty-eight screws (97.7%) were placed and analyzed in this study. One screw was skipped due to limited arm-reach of the robot and another due to clamp position blocking the screw trajectory. Screw tip, mid-pedicle, and tail positions and angular accuracy are summarized in Table 1.

**Table 1:** Accuracy of pedicle screws (*N* = 88)

	Tail ML (mm)	Tail AP (mm)	Tail SI (mm)	Tail 3D (mm)	Mid-pedicle ML (mm)	Mid-pedicle SI (mm)	Mid-pedicle 2D (mm)	Tip ML (mm)	Tip AP (mm)	Tip SI (mm)	Tip 3D (mm)	Angle (degrees)
Systematic Error (SME)	0.02	− 0.83	− 0.64	N/A	0.19	− 0.57	N/A	0.45	− 0.85	− 0.49	N/A	1.58
Precision (2 SDs)	2.24	2.32	1.09	N/A	1.79	0.84	N/A	1.61	2.31	1.09	N/A	1.61
Accuracy (MAE)	0.90	1.06	0.68	1.78	0.75	0.60	1.04	0.77	1.06	0.62	1.67	1.58

*ML* = medial(+)/lateral(−), *AP* = anterior( +)/posterior(−), ***SI*** = superior( +)/inferior(−), *3D* three dimensional, i.e., Euclidean error, *2D* two dimensional, i.e., Euclidean error in the coronal plane, *Angle* composite angular error. *SME* signed mean error, *SD* standard deviation, *MAE* mean absolute error

**Fig. 3:**
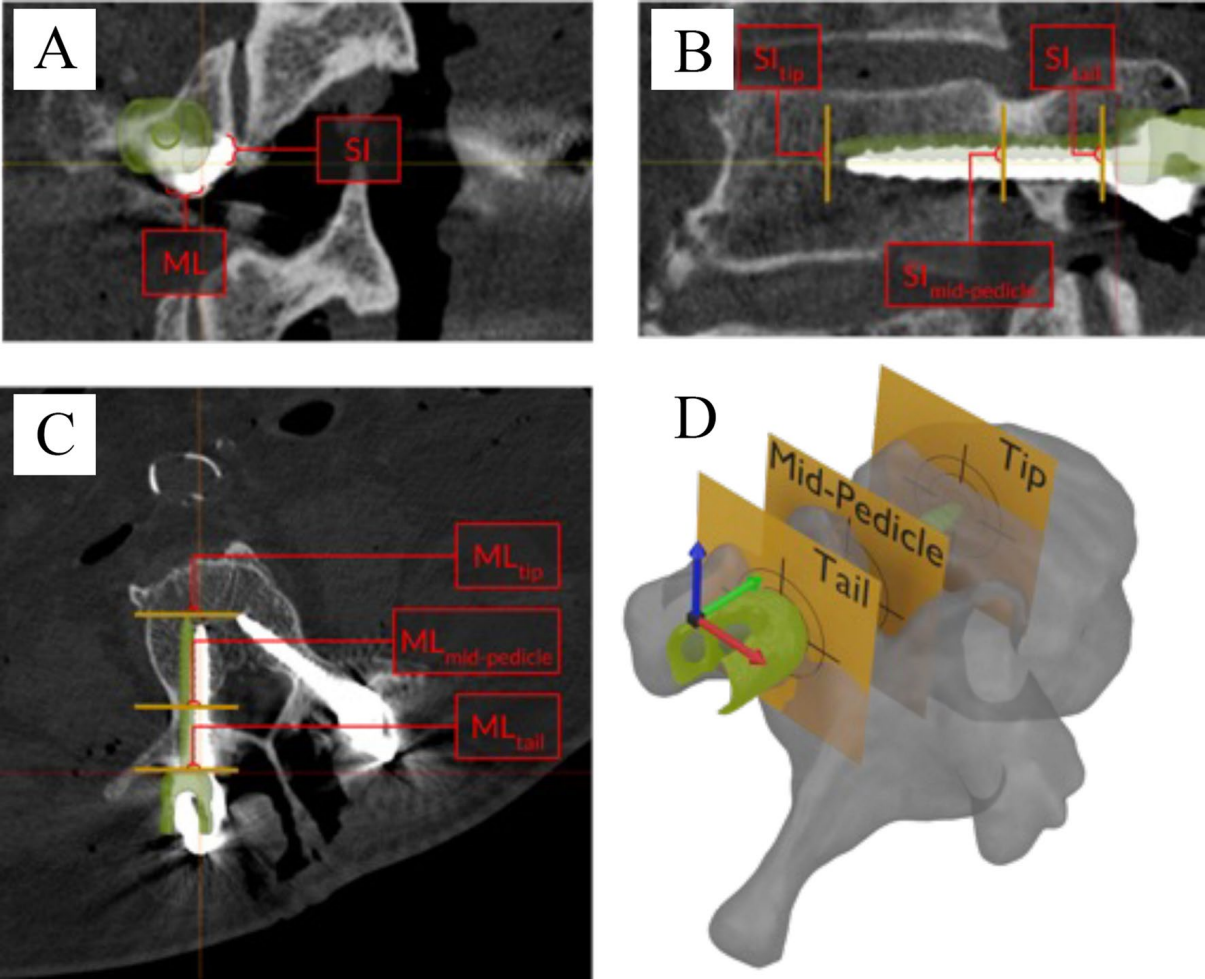
Axis-aligned slices of postoperative CT images showing simulated error calculations. Analysis planes (tail, mid-pedicle, and tip) are shown in yellow and the preoperative planned screw position is overlaid in green. Errors are labeled ML for medial–lateral, SI for superior–inferior; in this case, all ML errors are positive (medial) and all SI errors are negative (inferior) **A**: coronal imaging, **B**: sagittal imaging, **C**: axial imaging **D**: 3 dimensional representation of the vertebrae demonstrating the tail (entry point of the screw), mid-pedicle (point the screw is closest to the pedicular wall, and screw tip

The mean Euclidean error at the tip and tail were 1.67 mm and 1.78 mm, respectively. The Euclidean error at the mid-pedicle position was 1.04 mm; 2D analysis is utilized since “depth” of screw penetration was not a factor in the mid-pedicle location (Fig. 3). Figure 3A–C provides a graphical presentation of these data as “target analysis” for the tip, tail, and mid-pedicle positions.

Screw accuracy is reported by regions in Table 2. The most clinically consequential measurement is the medial–lateral mid-pedicular absolute error; one-way ANOVA demonstrates significant differences in this value between regions. Post-hoc pairwise tests showed that upper thoracic and lumbar regions are more accurate than lower thoracic (*p* < 0.03 and *p* < 0.01, respectively).

**Table 2:** Pedicle screw accuracy by region

	Tail ML (mm)	Tail AP (mm)	Tail SI (mm)	Tail 3D (mm)	Mid-pedicle ML (mm)	Mid-pedicle SI (mm)	Mid-pedicle 2D (mm)	Tip ML (mm)	Tip AP (mm)	Tip SI (mm)	Tip 3D (mm)	Angle (degrees)
Upper thoracic (T2–T6) pedicle screws (*N* = 30)												
Systematic error (SME)	− 0.06	− 1.24	− 0.50	N/A	0.15	− 0.52	N/A	0.43	− 1.25	− 0.54	N/A	1.44
Precision (2 SDs)	1.85	2.32	0.95	N/A	1.58	0.68	N/A	1.64	2.32	0.82	N/A	0.86
Accuracy (MAE)	0.74	1.27	0.57	1.77	0.64	0.55	0.91	0.78	1.29	0.58	1.84	1.44
Lower thoracic (T7–T12) pedicle screws (*N* = 34)												
Systematic error (SME)	0.08	− 0.59	− 0.56	N/A	0.24	− 0.49	N/A	0.52	− 0.62	− 0.40	N/A	1.83
Precision (2 SDs)	2.87	2.19	1.06	N/A	2.27	0.88	N/A	1.89	2.17	1.31	N/A	1.07
Accuracy (MAE)	1.18	0.95	0.59	1.84	0.97	0.53	1.17	0.91	0.95	0.65	1.67	1.83
Lumbar (L1–L4) pedicle screws (*N* = 24)												
Systematic error (SME)	0.04	− 0.65	− 0.92	N/A	0.18	− 0.76	N/A	0.39	− 0.66	− 0.54	N/A	1.39
Precision (2 SDs)	1.66	2.31	1.14	N/A	1.25	0.88	N/A	1.10	2.32	1.07	N/A	0.69
Accuracy (MAE)	0.69	0.94	0.96	1.70	0.56	0.76	1.00	0.56	0.94	0.64	1.45	1.39

*ML* = medial(+)/lateral(−), *AP* = anterior(+)/posterior(−), ***SI*** = superior(+)/inferior(−), *3D* three dimensional, i.e., Euclidean error, *2D* two dimensional, i.e., Euclidean error in the coronal plane, *Angle* = composite angular error. *SME* signed mean error, *SD* standard deviation, *MAE* mean absolute error

On visual CT evaluation, 78 (88.6%) of screws were identified as Grade A and 10 (11.4%) identified as Grade B. Direct visualization after laminectomy revealed 3 breaches (3.4%), all less than 2 mm (Grade B). Two of the three breaches identified upon open dissection were also identified on the CT. The third breach was not identified on CT and was scored as Grade A by both raters (33.3% false-negative rate). CT assessment resulted in eight false-positive breach identifications when open dissection was used as the gold standard (8.4% false-positive rate).

Evaluation of registration accuracy was limited by percutaneous placement of the tantalum beads, with only 8 of the 16 beads were available for assessment due to either screw overlap or extra-osseous placement of the beads. Assessment of registration accuracy revealed the positional MAE ± SD was 0.45 mm ± 0.42 mm. The perturbation analysis found a mean translation error of 0.05 mm ± 0.05 mm and a rotation error of 0.07° ± 0.08°.

## Discussion

This study is the first description of a fully automated algorithm for determination of the technical accuracy of pedicle screw placement that does not require human intervention in the rigid-body alignment or measurement process. The technical accuracy results from this study of the Mazor X Stealth robotic system are equivalent to or better than reported accuracy from any prior in vivo or cadaver study [5, 19, 20, 26, 27, 32, 33, 38–42]. At the mid-pedicle position, our MAE was 0.75 mm in the ML direction, 0.60 mm in the SI direction, and 1.04 mm in total. Our total angular error of 1.58° was also superior to any prior cadaver or in vivo study [5, 19, 26, 27, 29, 32, 33, 38–42].

Prior to the development of technical accuracy measurement systems, the G–R classification system served a useful role providing categorical accuracy and safety data. If we accept the premise that Grade A and Grade B are clinically acceptable, then only computer-assisted techniques (freehand navigation and robotically assisted navigation) consistently achieve the goal of 100% Grade A and B results (Table 3) [5, 19, 20, 26, 27, 32, 33, 38–42]. The current cadaveric study supports this conclusion, with all screws identified on CT as Grade A (88.6%) or B (11.4%), although only 3 screws were confirmed as breached after open dissection (8.4% false-positive rate).

**Table 3:** Summary of prior accuracy studies

*Studies*	Technique			Spine levels	# of screws	G–R class (%)						2D screw tip error (mm)	2D screw tail error (mm)	3D screw tip error (mm)	3D screw tail error (mm)	Mid-pedicle error (mm)			3D total angular error (°)
	FH	Nav	Robot			A	B	A + B	C	D	E					ax	sag	Total 2D	
Cadaver/bone model studies																			
** Groisser** **2024**			**Mazor 3rd Gen**	**T2–L4**	**88**	**88.6**	**11.4**	**100**				**1.23**	**1.07**	**1.78**	**0.79**	**0.75**	**0.6**	**1.07**	**1.58**
Frisk 2022		Nav		T11–L4	48	72.9	20.8	93.7	6.3			1.4	1.9						3.00
Vaccaro 2020	MIS			T10–L5	160	60	20	80	10	3	8	No summary data reported							
			Globus MIS			97.5	3	100											
	Open					52.5	15	67.5	15	8	10								
			Globus Open			92.5	8	100											
Lieberman 2012	Fluoro			T9–S1	190	54.1	32.4	86.5	8.1	5.4				2.59					
			Mazor			66.2	26.2	92.4	6.1	1.5				1.12					
Human in vivo studies																			
Ha 2023			CUVIS-Spine	T11–S1	448	88.4	9.6	98	1.56	0.22	0.22	2.48	2.86						
Volk 2023			Mazor 3rd Gen	Lumbar	500	71.2	26	97.2	1.6	0.01	0.01					1.75	1.52		3.07
Benech 2022			Globus	T11–S1	726	84.6	13.4	97.9	1.7	0.4		1.9	2.2						2.9
Toosi 2022			Globus	T12–S2	2946	84.6	13.2	97.8	2	0.2		1.75	1.78						2.25
Kanaly 2022			Globus	L1–S1	326	82	15.5	97.5	1.5	1		1.9	2						2.6
Gubian 2022		Nav		L1–S1	140	92.1	7.9	100						5.5	5.2				6.3
Wallace 2020			Globus	T1–S2	600			98.2	1.5	0.3		1.7	1.8						2
Jiang 2020			Globus	T1–S2	254	72.4	27.6	100				3.6							3.6
Han 2019	Fluoro			Thoraco-lumbar	585	95.3	3.4	98.7	0.9	0.4									
			Ti		532	86.1	7.4	93.5	4.6	1.4	0.5			1.6	1.4				

The bolded text is the data from this present study

*G–R Class* Gertzbein–Robbins Classification, *2D* two dimensional, *3D* three dimensional, *FH* freehand, *Nav* computed tomography-guided navigation, *Robot* robotic navigation, *ax* axial, *sag* sagittal, *MIS* minimally invasive surgery, *Open* open surgery, *Fluoro* fluoroscopy

The difficulties assessing pedicle breach and differentiating between Grade A and Grade B screws secondary to scatter cannot be underestimated, as well as observer bias and lack of a standardized approach to assessing the CT scans. It is likely that prior studies of breach based upon CT evaluation may also suffer from high false-positive rates; however, it is most likely to impact review of pedicle screws with less than 2 mm of breach, which are generally deemed clinically acceptable.

It is difficult to assess the technical accuracy of freehand techniques since freehand surgeons generally do not obtain preoperative 3D imaging or plan their screws on 3D planning software. However, a recent non-consecutive retrospective study of freehand pedicle screw categorical accuracy in 318 pediatric spinal deformity patients with 6,358 screws reported 2.63% of the pedicle screws were Grade C or worse, and 0.26% of screws necessitated UPROR [6]. Another non-consecutive multicenter retrospective review reported 0.26% incidence of both neurologic injury and misplaced instrumentation [7]. A single-center retrospective review of all pediatric patients who underwent spinal fusion over a 30-year period revealed a 1.1% incidence of UPROR related to malpositioned pedicle screws, neurological changes, or pneumothorax (presumably related to implants) [43]. Meta-analyses report overall accuracy rates between 90.6% and 94.9% for freehand and freehand navigation techniques [44, 45]. Collectively, these studies provide useful baseline data for accuracy and revision rates for pedicle screws using non-robotic techniques. The retrospective and non-consecutive nature of the pediatric multicenter database studies limit their utility for comparison with consecutive series of computer-navigated surgical accuracy in vivo and single-center consecutive case reviews [5, 19, 20, 26, 27, 32, 33, 38–43].

The advent of computer-assisted surgical navigation has fostered the development of technical accuracy, namely geometric comparisons between the 3D preoperative CT plan and the postoperative CT scan. Freehand navigation and robotically assisted navigation studies have variably described the technical accuracy of pedicle screw placement with increasing sophistication, but without any consensus on terminology or analysis methodology [5, 13–17, 19, 20, 22, 26–29, 32, 33, 38, 40–42]. The protocol described in this work is greatly influenced by a small number of studies utilizing either cadavers, bone models, or in vivo human studies which have iteratively improved the granular reporting of 3D technical accuracy (Table 3) [5, 19, 20, 26, 27, 32, 33, 38–42]. The largest studies of computer-assisted freehand and robotic navigation report total angular errors between 2.0 and 6.3 degrees compared with 1.58 degrees in the current study. Only one study by Volk et al. evaluated mid-pedicle error, which was 1.75 mm in the ML direction and 1.52 mm in the SI direction, compared to 0.75 mm and 0.60 mm in the current study [26].

**Fig. 4:**
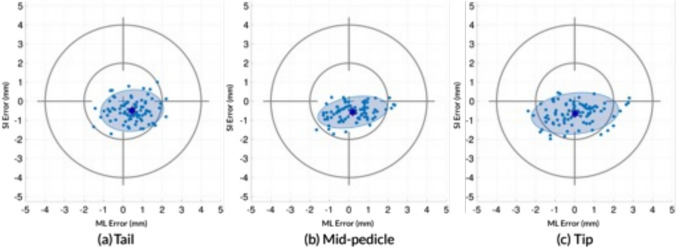
Coronal error at screw tail (**a**), mid-pedicle (**b**) and tip (**c**). The origin (center of the target) is the planned screw trajectory and the blue (circle) marks represent actual individual screw positions; right-side screws have been mirrored across the sagittal plane to standardize laterality. Purple stars indicate the signed mean error, while the shaded ellipse represents two standard deviations in screw position. Grey target circles show 2 and 4 mm coronal error relative to planned position. *ML* = medial(+)/lateral(−), *SI* = superior( +)/inferior(−)

A combination of standardized clinical safety data (e.g., G–R classification) and technical accuracy including precision is necessary to properly compare different navigation systems and workflows. We advocate a screw-aligned coordinate system as the natural reference frame to present useful feedback for surgeons, and we present “target analysis” visualizations (Fig. 4) to illustrate accuracy (mean error) and precision (2 SD) [19, 26, 33, 38, 40]. Although most prior authors reference screw tip and tail accuracy, we agree with Volk et al. that accuracy data at the mid-pedicle is most critical, as this describes proximity of the pedicle screw to the spinal canal [26]. Furthermore, in addition to MAE, we would strongly endorse reporting SME, as this provides valuable information about directionality of systematic errors and allows direct comparison between screws and across studies. Similarly, the variance (or SD) of the SME indicates the precision/consistency of the surgical technique.

A limitation of prior accuracy studies is the requirement for human experts to perform the overlay of the preoperative plan with the postoperative screw position. This study reports the development of a fully automated protocol for determination of pedicle screw accuracy utilizing standard preoperative and postoperative CT scans. Our reported registration accuracy (0.48 mm) was limited by the imaging resolution (0.625 mm), while perturbation analysis showed the algorithm to be extremely consistent. In future studies, we intend to reduce the uncertainty of our registration accuracy, and we believe such assessments should be a prerequisite for technical accuracy data reporting. Some limitations of the current study include the small number of pedicle screws assessed, as well as the use of cadavers instead of in vivo screw assessment. The cadavers did not have any spinal deformity, which likely would impact accuracy results. We recognize that only a subset of accuracy errors in pedicle screw placement result in patient harm. However, improvements to accuracy, precision, and reliability of pedicle screw placement using robotically assisted surgical navigation have the potential to reduce the incidence of patient harm by decreasing the 0.26% incidence of UPROR secondary to malpositioned pedicle screws [6, 7, 43]. A standardized, systematic approach to the reporting of pedicle screw accuracy with computer-assisted pedicle screw insertion techniques using standardized nomenclature as well as a screw-centric 3D coordinate system is vital. Standardized cadaver and human models will greatly facilitate testing, comparison, and improvement of robotic systems (ASTM F2554-18).

## Data Availability

No datasets were generated or analysed during the current study.
